# ‘The palliative care ambulance’: A qualitative study of patient and caregiver perspectives of an ambulance service

**DOI:** 10.1177/02692163231166760

**Published:** 2023-04-24

**Authors:** Aileen Collier, Ann Dadich, Cathie Jeffs, Andrew Noble, Gregory B Crawford

**Affiliations:** 1 Research Centre for Palliative Care Death and Dying (RePaDD), Flinders University of South Australia, Adelaide, South Australia; 2 Northern Adelaide Local Health Network, South Australia, Australia; 3 School of Medicine, Faculty of Health and Medical Sciences, The University of Auckland, Auckland, New Zealand; 4 School of Business, Western Sydney University, Penrith, Australia; 5 South Australian Ambulance Service, South Australia, Australia; 6 College of Medicine and Public Health, Flinders University of South Australia, Adelaide, South Australia; 7 Adelaide Medical School, Faculty of Health and Medical Sciences, University of Adelaide, Adelaide, Australia; 8 Northern Adelaide Palliative Care Service, Northern Adelaide Local Health Network, South Australia, Australia

**Keywords:** Community-based palliative care, emergency medical technicians, palliative care, primary health care

## Abstract

**Background:**

The need for home-based palliative care is accelerating internationally. At the same time, health systems face increased complexity, funding constraints and global shortages in the healthcare workforce. As such, ambulance services are increasingly tasked with providing palliative care. Where paramedics with additional training in palliative care have been integrated into models of care, evaluations have been largely positive. Studies of patient and family carer experiences of paramedic involvement, however, are limited.

**Aim:**

To explore patient and family caregiver experiences of paramedics’ contribution to palliative care at home.

**Design:**

Qualitative interview study. We analysed data within a social constructionist epistemology using reflexive thematic analysis.

**Setting/participants:**

Participants receiving specialist palliative care in the community of a metropolitan city of Australia who requested an ambulance between January and August 2018, inclusive.

**Results:**

Participants considered paramedics with expertise and experience in palliative care as an extension of the specialist community palliative care team and held them in high regard. Participants highlighted the importance of: critical palliative care at home and a timely, responsive approach; person-centred paramedics; as well as safety and security.

**Conclusion:**

Patients and carers feel safe and secure when they know that highly responsive skilled professional support is available when an unexpected problem or sudden change arises, especially out-of-hours, and that support is delivered in an empathic and person-centred manner.

**What is already known about the topic?**

- As death approaches, presentations to emergency departments can increase. Despite being a priority for patients and families, there are significant gaps in out-of-hours palliative care. The role of paramedics in providing out-of-hours palliative care has expanded. However, little is known about patients’ and families’ experiences.

**What this paper adds?**

- This study shows that patients and carers recognised paramedics as critical members of the community specialist palliative care team, rather than a separate emergency service. Patients and carers value paramedics’ timely response; their person-centred approach; and the safety and security they provide.

**Implications for practice, theory or policy**

- Future research addressing the cost-effectiveness of community care models should include paramedics as well as outcomes of safety and quality of care
- Paramedic education programmes should consider values and attitudes towards death and dying, as well as loss and grief
- It is also important to evaluate the education, training and ongoing support for paramedics to optimise their capacity to deliver palliative care

## Introduction

The demand for palliative and end-of-life care at home is growing at a rapid rate, internationally. For instance, the annual number of deaths in Australia is expected to double in the next 40 years.^1^ The need for 24-h care for people with palliative care needs living in the community will significantly increase. People in the last year of life are high users of unscheduled care.^2^ Simultaneously there is a global healthcare workforce shortage.^3^ For example, there has been a sharp decline in community nurses in the United Kingdom, despite the increasingly complex needs of people requiring palliative care. In the United Kingdom, only 25% of community nursing teams reported sufficient staffing to meet patient needs.^4^ Almost 30% of district nursing teams in the United Kingdom have a caseload of over 400 patients.^5^ Furthermore, a recent Scottish study highlighted that people with organ failure were less likely to be identified in primary care as requiring coordinated care, relative to other groups who require palliative care – and ambulance and emergency departments were the unscheduled services they used most.^6^

Despite out-of-hours care being a significant component of palliative care at home, most resources tend to be directed to in-hours care. A recent report from the United Kingdom demonstrated that out-of-hours care is fragmented and inadequate.^7^ Likewise, a recent systematic review failed to identify studies that evaluated the effectiveness or cost-effectiveness of adult out-of-hours specialist or generalist palliative care.^8^ In the United Kingdom and Ireland, the palliative and end-of-life care priority setting partnership prioritised the need to identify the best ways to provide palliative care outside of working hours to avoid crises and help patients to stay in their place of choice.^9^

Historically, paramedics have focussed on life-saving and curative care. However, more people are likely to face an uncertain illness trajectory due to complex and chronic illnesses.^10^ A recent Australian survey over 12 months reported that 0.5% of the ambulance caseload was related to people experiencing a health crisis associated with palliative care. Paramedics recorded the primary assessment as pain, respiratory symptoms and death. Approximately three-quarters of the palliative care patients who contacted the ambulance service in this study were transported to hospital.^11^ Paramedics have reported unmet educational needs, concerns about legal issues, and limited knowledge of goals of care and treatment plans.^12,13^

Notwithstanding the aforementioned issues, there is increasing recognition that paramedics have an important role in managing acute symptoms and supporting patient and carer preferences in terms of care goals and place of care and death.^14^ As a mobile healthcare workforce, they are well-placed to respond to patients requiring unscheduled care and can be an overlooked resource in community-based palliative care.^15^ When care providers cooperate, patients can be cared for and able to die at home when they might otherwise have been cared for or died in hospital.^16^ For example, when French paramedics collaborated with community-based palliative care networks, patient care goals were more likely to be met.^17^ Collaborations between two ambulance services and Macmillan Cancer Support in the United Kingdom^18^ and a similar collaboration in Australia have demonstrated how paramedics contribute to palliative care at home. A recent Finnish study found that the systematic integration of paramedics into end-of-life care at home using a planned protocol was reasonable, but that research is needed to evaluate patient and carer perspectives.^19^ Canadian paramedics contribution to palliative care was viewed positively as part of a wider evolution of the professional paramedic role in response to health system challenges. Paramedics valued being able to provide care that was patient centred and meant transport to hospital was not always necessary.^20^

Thus, the aim of this study was to explore patient and family caregiver perspectives of an ambulance service including extended care paramedics who had education and experience in palliative care.

## Methods

### Research design

This study clarifies the experiences of patients with palliative care needs and their carers with the ambulance service. This was achieved using a qualitative interview study design. A social constructionist epistemology underpinned this qualitative study, whereby qualitative interview data were analysed using Braun and Clarke’s^21^ approach to reflexive thematic analysis.

### Setting

#### Community palliative care in metropolitan Adelaide

The multidisciplinary community specialist palliative care service provides a consultative service to primary care providers, including general practitioners (GPs), district nurses and aged care facilities as part of the public health system in South Australia. The specialist service operates from 8:00 am to 5:00 pm, 5 days a week, with a nurse-led service on weekends (8:00 am–5:00 pm). A medical registrar provides 24/7 on-call telephone support, supported by an on-call specialist palliative care consultant. The state government contract community district nursing services. These are provided by a not-for-profit community nursing services. Referral is via a central point of triage for the whole of metropolitan Adelaide. Community nursing services are provided through a central referral unit, operating from between 8:00 am and 8:00 pm, 7 days a week and unable to attend to emergencies. In part, as a result of COVID-19, a specialist palliative care nurse practitioner provides a rapid response (Monday to Friday) in situations of urgent need or hospital discharge where the community nursing service and/or GP are unable to provide a response in a timely manner.

#### South Australian ambulance service extended care paramedics

The extended care paramedics programme (commenced in 2008) is a joint initiative of South Australia (SA) Health and South Australia Ambulance Service (SAAS). Extended care paramedics are intensive care paramedics who receive specialist intensive training, including specialist placements. They work in close collaboration with other healthcare professionals to manage and treat people in their usual place of residence in metropolitan and fringe areas of SA. They are available 24 h a day 7 days a week. Palliative care was added to their role in 2011, as a key initiative of the State-wide palliative care services plan in South Australia (SA)^22^ a rapid response programme to support patients and their family caregivers at home. They carry equipment to address palliative care needs of patients in the metropolitan area of SA^23^ and liaise closely with the specialist palliative care team. An extended care paramedic is always present in the emergency operations centre of SAAS as well as on the road.^24^ All callers to the emergency number are triaged through an emergency algorithm – however extended care paramedics can intervene at the point of triage to provide an alternative to hospital transport where deemed appropriate.

## Participants

### Population

Eligible participants were individuals who: were adult patients with palliative care needs, as determined by a clinician involved in their care; and self-nominated family carers. Furthermore, they had to be: aged 18 years or over; conversant in English; and able to provide informed consent.

### Sampling

We purposively sampled anyone with palliative care needs who had at least one contact with an extended care paramedic in the catchment of the local health network. Sample size was underpinned by ‘information power’.^25^ That is, our analysis was driven by diversity of participants’ experiences along with the associated depth of the data in relation to our research question.

### Recruitment

Participants were recruited using ambulance service records and via the team case lists of one of metropolitan Adelaide’s three specialist palliative care teams. A member of the specialist palliative care team invited eligible participants to contribute to this study between January and August, 2018 (inclusive) – this team member helped to identify those who might have had experience with the ambulance service. The team member contacted eligible participants, asking if they could be contacted by a researcher. Following consent, a researcher contacted the eligible participant to invite their participation in a semi-structured interview at a time and place of their choosing. A multisite ethics research committee approved the study. Research governance was obtained from the Northern Adelaide Local Health Network and the South Australian Ambulance Service.

### Data collection

Participants were provided the opportunity to be interviewed: face-to-face or via telephone; and together (patients and family carers) or individually. Two researchers (AC and CJ) conducted the interviews. All interviews occurred face-to-face in the patient’s home, except for one telephone interview. Because participants might not have distinguished different ambulance personnel, they were asked to describe their experiences with the ambulance service, including the role of any ambulance officer and/or extended care paramedic in their care – this was aided with the question: ‘Tell me about your experience of calling an ambulance’ and/or where the participant knew of the extended care paramedics, they were asked to tell us more about that experience. Reflecting extant literature,^26^ skilled researchers used clarifying questions, prompts and cues, according to participant narratives. For example, the researchers asked, ‘Can you tell me what or who prompted you to call for an ambulance? What did you hope the ambulance would do?’ When a family carer expressed, ‘The paramedic was amazing’ or similar, the researcher responded, asking, ‘Tell me what made them amazing’. Interviews lasted between 50 and 67 min and participants were provided the opportunity to have their interview digitally-recorded.

### Data analysis

Data analysis commenced during data collection using a reflexive thematic approach.^21^ Field researchers were both palliative care nurses: one nurse was employed within the palliative care service and had no clinical relationship with participants. The other was an experienced academic researcher. They noted their reflections about the interviews as they happened and in relation to previous interviews. As the study progressed, interviewers reflected with the study team (a palliative care physician researcher and a researcher with a background in psychology and experienced in organisations and knowledge translation) on what these data inferred about meaningfulness in how patients and family caregivers experienced the role of ambulance paramedics in palliative care. The first author coded data in relation to these discussions and generated initial themes. These themes were developed with the research team, who include a palliative care physician and senior researcher, a senior psychology researcher with expertise in implementation science, and an experienced extended care paramedic clinical lecturer. In keeping with our reflexive approach to thematic analysis and our starting place, we viewed our combined research and clinical experience as a resource in the analytical process – as Braun and Clarke stated: meaning and knowledge are understood as situated and contextual, and ‘researcher subjectivity is conceptualised as a resource for knowledge production, which inevitably sculpts the knowledge produced, rather than a must-be-contained threat to credibility’ (pp. 334–335).^27^ This process of latent ‘open’ coding and theme development evolved throughout the analytical process to gain rich interpretations of patterns of meaning in the data.^28^ That is, we sought to identify implicit and underlying meaning in our process of open coding, focusing on what matters most to patients and families.

## Findings

Data were generated from patients (*n* = 6), and family carers (*n* = 11; see Table 1). One patient ID2 was in the terminal phase. The researcher interviewed all participants in person, together in their home. Our analysis generated the following key themes: critical palliative care at home and a timely, responsive approach; person-centred paramedics; as well as safety and security.

**Table 1.** Participant characteristics.

	*n*
Age range	
45–54	1
55–64	1
65–75	3
>75	2
Gender	
M	4
F	3
Primary diagnosis	
Metastatic cancer	3
End stage heart failure	1
Stroke	1
COPD	1
Unknown	1
Birth country	
Australia	4
Other	3
Number of family participants	
0	1
1	2
2	3
3	1

### Critical palliative care at home: A timely, responsive approach

Participants frequently conveyed instances of an extended care paramedic who responded to an immediate need when other service providers were not immediately available, including community health nurses, palliative care specialists and general practitioners. For instance, a wife whose husband had end-stage pancreatic cancer conveyed how the extended care paramedic supported her ‘out-of-hours’ when she ran out of breakthrough syringes of analgesia:

Patient’s son: It was. . . Thursday night before Easter. We ran out of breakthrough for dad. Mum rang to the get the district nurse out – ‘Sorry, everyone’s gone home; we’ll get an ambulance for you’. So, the ambulance came over and he drew up some breakthroughs for Dad. . . it made it easier for the night, not having him in pain all night.

Patient’s wife: It was the palliative ambulance. He was the palliative ambulance guy. I apologised and I said, ‘I’m so sorry for getting you out’ and. . . [the extended care paramedic] said ‘Don’t apologise’, and I said. . . ‘[The district nurses] have got the policy – throw out the breakthroughs 24 hours after. And. . . [the patient] was going through about six to seven a day and they just threw out my fresh batch’. . . I just had a really bad day. I had a shit of a day and so having him come in and [say], ‘Never apologise. If you need us, you just ring us and ask for’, and he gave me the name. . . ‘If we don’t answer straight away, we will get back to you and don’t leave it too long’, because I only had two breakthroughs left and [my husband]. . . was in bed and I said I have to ring someone and so that’s how we got the ambulance. . . here and he made them up – [the syringes with breakthrough analgesia] (ID6).

This quote shows how the extended care paramedic was not only able to address the issue of the breakthrough analgesia, but alleviated this wife of a patient’s frustration at having to throw away her husband’s medication, leading to her concern about not having enough doses of breakthrough medication to get through the night.

Participants emphasised the time ambulance personnel spent with them and their efforts to prevent hospital admissions. Furthermore, they valued knowing that the ambulance team had done everything possible to keep a patient at home, even when a hospital transfer was inevitable. A wife whose husband had dementia and metastatic prostate cancer was aware of the imperative of ‘hospital avoidance’, whereby many governments, including that in Australia and the United Kingdom,^29,30^ discouraged hospital admissions and encouraged community-based care. Nevertheless, the healthcare imperative to free-up beds was aligned with her own preference for her husband to be cared for at home:

Patient’s wife: it was a normal call-out and then. . . [the paramedic] was the one that got. . . his boss. . . he said, ‘there’s something wrong’. . . so they called in this other ambulance and they waited till he came; then the other guy came. He was in a. . . [sport utility vehicle]. . . and he got the ambulance lady to reinsert. . . [the catheter]. She put a fresh one in, but it wouldn’t stop bleeding; so he said, ‘What we’ll do is get two ice cold cloths and we’ll try and stop the bleeding’. So, I found two cloths, put one in the freezer and ran the other one under the tap and then he said, ‘I think we need another one.’ So, we got the other one and. . . he said, ‘Look, I’ve bandaged it. I’m sorry you gotta go in [to hospital]’. This was after about three hours. So, he tried his damnedest to be able to stay at home. . .

Researcher: What did you want?

Patient’s wife: I just wanted him fixed – actually, I would have preferred him to stay home. Anyway, we ended. . . in [hospital] (ID 5).

Although not always recognised or legitimised, the extended care paramedics were integral members of the multidisciplinary specialist palliative care team. When a palliative care medical registrar received an out-of-hours call from a patient at home, they frequently relied on the extended care paramedic to attend onsite to address a patient and daughter’s concerns:

Patient’s daughter: Pain in his stomach – he hadn’t been able to do a wee – I said, ‘Mum, I’m coming over’ – so, I rang the number that I was given from the district nurse from the folders that they’ve got and then I rang triple zero [the emergency telephone number] and asked to speak to the extended care people. But when I was on the phone, I spoke to a [palliative care doctor]. . . she was great – that was about ten o’clock. . . that was excellent because we talked about different options. So, then we rang the ambulance. . .

Researcher: And she advised you to call the ambulance?

Patient’s daughter: Yeah. . . so we could get. . . a catheter put in, some subcut[aneous] pain relief and then be on our way. But when we rang, I was instructed that the extended care paramedics were unavailable, which was a real shame. . . when the two. . . [ambulance crew] did arrive, they were unable to put a catheter in, unable to give subcut[aneous] pain relief and then we rang the doctor back and we made a decision for him to go into hospital and we wanted him to come back [home] straight away – so just go to emergency for a catheter and some pain relief (ID2).

This quote shows how the extended care paramedic contributed to the continuity of patient care – they were an expected extension of the specialist palliative care service in the delivery of out-of-hours care. Their level of skill and responsiveness was such that their unavailability on this occasion resulted in a visit to the emergency department.

### Person-centred paramedic care

Participants frequently expressed how extended care paramedics were responsive to their individual preferences and needs in terms of communication, information-giving, continuity of care and inclusion of family caregivers. They articulated how extended care paramedics often went above and beyond, sometimes enacting a work around to meet these needs. For one wife, ensuring her husband saw his own oncologist at the private hospital demonstrated high-quality person-centred care:

Patient’s wife: We packed all [the patient’s]. . . stuff up and things and this was all about nine am and they checked him out and yes. . . [they] want[ed] to go to hospital. . . we packed everything up and. . . the. . . [ambulance crew] actually took the long way because they couldn’t take him and leave him there [in the private hospital] and the doctor wasn’t gonna be there till ten. They got to the hospital at ten and they put themselves out to check and ring. . . [the oncologist] because that’s where. . . [my husband]. . . wanted to go – he needed to go to hospital, but he wasn’t an emergency and he needed to have his doctor there because it was to do with his cancer and I thought that was absolutely brilliant. . . [my husband] was happy and because he was happy, I was happy (ID3).

For some participants, the paramedics’ thorough assessments and the time taken to communicate with them, was highly valued. For instance, a patient’s daughter conveyed how she was unaware that extended care paramedics existed and how much she appreciated their availability particularly on a public holiday weekend:

Patient’s daughter: [The extended care paramedics were] awesome. . . [The paramedic] was here for two hours and she just sat here and we spoke, she checked out mum, she was on the phone to. . . [the] palliative care [service] and explained what was going on because it was Easter long weekend, so. . . [the palliative care service was] closed. . .. just her having the time; it wasn’t a race. . . in and out. And seeing how much time. . . just sitting there. . . talking to mum. It was absolutely awesome (ID 7).

### Safety and security

The availability and access to an extended care paramedic with palliative care skills at any time of day provided a feeling of safety and security for patients, family caregivers and healthcare professionals who were not on-call. They valued the psychological safety net that the extended care paramedic provided, knowing they were always available:

Patient’s daughter: I can call them anytime I want and they said, ‘Even if it’s nothing really and we come out and she’s okay, it doesn’t matter’. . . especially in the middle of the night, if something does go wrong. Because I called a. . . [general practitioner] to come out when I told them the symptoms that she had; they said: ‘We can’t come out’. Palliative care told me to ring the. . . [general practitioner], ask them to come out, and then contact them and tell them what’s going on and then I rang palliative care and said they won’t come out and she goes: ‘What?’. . . [I told the palliative care service] ‘They just said call the ambos’. . . that’s when [the palliative care service]. . . gave me the number for the extended care paramedics. . . I think I prefer them out than a [general practitioner] (ID7).

As the aforesaid quote suggests, extended care paramedics were often considered to be a source of out-of-hours support, even if a clinical intervention was not required. Carers juxtaposed other services and clinicians, who were often unavailable, with the paramedics who were both available and accessible. In addition to being available out-of-hours, the paramedics were also approachable – this was partly due to their warm and affirming nature, reaffirming carers’ incredible efforts:

Patient’s wife: [The extended care paramedic] made me feel good. . . because. . . he said, ‘Follow your gut instincts – you’re doing a darn good job of looking after your husband’ and even the kids – he even praised [my son]. . . because. . . [he] has been through a hell of a lot too. . . I actually got a big cuddle out of [the extended care paramedic]. . . he said, ‘You keep going; you’re doing a good job’ (ID 6).

## Discussion

### Main findings

This study shows that from participants’ perspectives, extended care paramedics were critical to the provision of out-of-hours home-based palliative care. Participants saw extended care paramedics as highly responsive outreach professionals and an extension of the specialist palliative care team- rather than emergency workers operating in isolation. As well as technical task focussed care, participant’s view of extended care paramedics was one closely aligned to palliative care philosophy.^31^ That is, they met emotional spiritual, social and psychological needs as well as technical care needs. Family carers particularly appreciated extended care paramedics’ supportive communication, the reassurance and affirmation of the care they provided, as well as the time ambulance personnel spent with them. This meant that family caregivers felt safe and secure in their caring role.

### What this study adds

Our study findings provide an important window into what is most important to patients and family caregivers to be able to stay at home when issues arise ‘out-of-hours’. In particular they highlight how important it is for family caregivers to know they can call on highly-skilled professionals to address their concerns at any time of day or night. This is consistent with participants in a New Zealand study who valued having a service that was able to respond any time of the day or night.^32^ Our findings support and extend the early evaluation of the SA paramedic model that patient and family caregivers level of satisfaction with extended care paramedics was high.^23^ Our data also support recent findings from Canada whereby service providers felt paramedics’ new palliative care role contributed significantly to family caregivers’ security and confidence in providing care at home.^20^

Our findings support those of a recent Canadian programme in which paramedic training resulted in high patient and carer satisfaction, as well as increased confidence in paramedics.^33^ Despite the imperative to deliver equitable, safe and high-quality palliative care to people at home, care systems around the world face significant workforce challenges. However, rather than simply fill a workforce or organisational gap, our data show that the extended care paramedics’ expertise, experience and skills along, with the capacity to respond rapidly, contributes to at-home palliative care in a way that other generalist services, such as GPs and district nurses, are unable. This could be partly because paramedics are experienced and trained in evaluating and responding to situations in the context of high uncertainty and complexity. For example, experienced paramedics are expert at applying communication skills in what are often emotionally-demanding, critical situations to gather information from patients, carers and bystanders.^34^ Our finding that extended care paramedics go above and beyond and sometimes enact positive deviance to do so, reflects Torabi and colleagues’ research.^35^ They found that, if ambulance personnel are not confident that a person will receive appropriate care upon entering the healthcare systems they are more likely to solve a problem by deviating from protocols.

### Implications for practice, policy and research

Our study highlighted the value participants placed on extended care paramedics in being able to address unexpected issues at home. The study of a national incidence reporting database in England and Wales found that almost 12% of reports pertained to patients and caregivers who were unable to access the care they needed, including access to medications. A further 10% pertained to incidents involving insufficient treatments (e.g. urinary catheters, pressure ulcer relief, nasogastric tubes) during a night or weekend, significant delays in commencing treatment, or the absence of necessary treatment.^36^ Our findings have important implications for how community palliative care service models are designed and how unscheduled care is operationalised.to meet the aforementioned unmet needs.

Despite numerous innovations to provide responsive ‘out-of-hours’ palliative care, service provision is highly variable. Future research addressing the cost-effectiveness of ‘out-of-hours’ community care models should include paramedics as well as other disciplines along with safety and quality care outcomes. If the role of paramedics extends to palliative care, paramedic education programmes will need to consider their values and attitudes towards death and dying, as well as loss and grief as well as symptom management at the end of life. The evaluation of continuing education models and undergraduate curricula for paramedics and how best to mentor and support them in palliative care practice are also important.

### Strengths, weaknesses, limitations of the study

We collected data from a metropolitan area in SA with its particular service model and associated culture. We do not claim our findings are necessarily transferable to other geographical areas. Our data predate the pandemic and thus findings might be different in the context of COVID-19 and the resulting additional pressures placed on health systems. Additionally, we are aware that participants might have self-selected as those who had a positive experience with extended care paramedics and other ambulance personnel. In addition, with the exception of a patient’s son, all family caregiver’s identified as women. Men may have a different perspective. Nevertheless, our findings show the value that patients and family caregivers place on the timely and skilled palliative care that extended care paramedics provide.
